# Lysyl oxidase‐like 2 is a regulator of angiogenesis through modulation of endothelial‐to‐mesenchymal transition

**DOI:** 10.1002/jcp.27695

**Published:** 2018-11-01

**Authors:** Olivier G. de Jong, Lizet M. van der Waals, Farah R. W. Kools, Marianne C. Verhaar, Bas W. M. van Balkom

**Affiliations:** ^1^ Department of Nephrology and Hypertension University Medical Center Utrecht, Utrecht University Utrecht The Netherlands; ^2^ Department of Physiology Anatomy and Genetics, University of Oxford Oxford United Kingdom; ^3^ Laboratory Translational Oncology, Cancer Center, University Medical Center Utrecht Utrecht The Netherlands

**Keywords:** angiogenesis, endothelial‐to‐mesenchymal transition, focal adhesion kinase, lysyl oxidase‐like 2, protein kinase B

## Abstract

Lysyl oxidase‐like 2 (LOXL2) belongs to the family of lysyl oxidases, and as such promotes crosslinking of collagens and elastin by oxidative deamination of lysine residues. In endothelial cells (ECs), LOXL2 is involved in crosslinking and scaffolding of collagen IV. Additionally, several reports have shown a role for LOXL2 in other processes, including regulation of gene expression, tumor metastasis, and epithelial‐to‐mesenchymal transition (EMT). Here, we demonstrate an additional role for LOXL2 in the regulation of angiogenesis by modulation of endothelial‐to‐mesenchymal transition (EndMT). LOXL2 knockdown in ECs results in decreased migration and sprouting, and concordantly, LOXL2 overexpression leads to an increase in migration and sprouting, independent of its catalytic activity. Furthermore, LOXL2 knockdown resulted in a reduced expression of EndMT markers, and inhibition of transforming growth factor‐β (TGF‐β)‐mediated induction of EndMT. Interestingly, unlike in EMT, overexpression of LOXL2 alone is insufficient to induce EndMT. Further investigation revealed that LOXL2 expression regulates protein kinase B (PKB)/Akt and focal adhesion kinase (FAK) signaling, both pathways that have been implicated in the regulation of EMT. Altogether, our studies reveal a role for LOXL2 in angiogenesis through the modulation of EndMT in ECs, independent of its enzymatic crosslinking activity.

## 1 INTRODUCTION

Lysyl oxidase‐like 2 (LOXL2) is a member of the lysyl oxidase family, which consists of lysyl oxidase (LOX) and lysyl oxidase‐like 1–4 (LOXL1–LOXL4). Like all lysyl oxidase family members, LOXL2 contains a conserved catalytic domain that catalyzes crosslinking of collagens and elastin by oxidative deamination of lysine residues (Kim, Kim, & Kim, 2011). Apart from its role in extracellular matrix (ECM) remodeling through crosslinking of collagens and elastin, LOXL2 also regulates a variety of intracellular signaling pathways involved in cell proliferation, differentiation, tumor metastasis, and epithelial‐to‐mesenchymal transition (EMT) (Ahn, Dong, & Oshima, 2013; Chang, Nicolau, & Cox, 2013; Herranz, Dave, & Millanes‐Romero, 2016; Iturbide, Pascual‐Reguant, & Fargas, 2015; Peinado, Portillo, & Cano, 2005).

The function of LOXL2 has been extensively studied in epithelial tumor cells; however, studies on the role of LOXL2 in endothelium are limited. We recently described that endothelial cells (ECs) secrete LOXL2‐containing exosomes that crosslink collagens in a hypoxia‐dependent manner (de Jong, van Balkom, & Gremmels, 2016; de Jong, Verhaar, & Chen, 2012). In line, Bignon, Pichol‐Thievend, and Hardouin (2011) showed that LOXL2‐mediated collagen crosslinking plays a regulatory role in endothelial cells sprouting and migration of endothelial cells. Interestingly, the authors showed that an enzymatic inhibitor of lysyl oxidases, β‐aminopropionitrile (BAPN), reduced the increase in sprouting after LOXL2 overexpression only by approximately 40%. Furthermore, BAPN reduced sprouting in human umbilical vein endothelial cells (HUVEC), but not to the same extent as a LOXL2 knockdown, strongly suggesting that LOXL2 regulates angiogenesis in part through processes independent of extracellular enzymatic activity.

Endothelial‐to‐mesenchymal transition (EndMT) is a process in which endothelial cells differentiate toward a mesenchymal phenotype. This process is characterized by a loss of apical‐basolateral polarity, increased ECM production, cell migration, sprouting, and an increase in mesenchymal marker expression, including α‐smooth muscle actin (α‐SMA). Furthermore, EndMT results in decreased expression of endothelial markers such as vascular endothelial cadherin (VE‐cadherin) and platelet endothelial cell adhesion molecule (PECAM‐1; van Meeteren & ten Dijke, 2012; Welch‐Reardon, Ehsan, & Wang, 2014). EndMT is a process that plays an important role in cardiovascular development and repair, fibrosis, and angiogenesis (Lin, Wang, & Zhang, 2012; Muylaert, de Jong, & Slaats, 2015; Welch‐Reardon et al., 2014), and is remarkably similar to EMT, a process in which epithelial cells undergo a differentiation toward a more mesenchymal, fibroblast‐like phenotype. Both processes are important in embryonic development and show a large overlap in regulatory pathways (Kovacic, Mercader, & Torres, 2012). Interestingly LOXL2 has been shown to play a regulatory role in EMT through a variety of mechanisms, including the inhibition of transcription factors that regulate both EMT and EndMT (Medici, Potenta, & Kalluri, 2011; Peinado, 2005). We thus hypothesize that, alongside its described role in ECM remodeling, LOXL2 regulates angiogenesis through modulation of EndMT.

## 2 MATERIALS AND METHODS

### 2.1 Cell culture

Human microvascular endothelial cells (HMEC‐1; Ades, Candal, & Swerlick, 1992) were maintained in MCDB 131 medium, containing 10% fetal calf serum (FCS), 100 U/ml penicillin, 100 µg/ml streptomycin, and 10 mM l‐glutamine (all from Thermo Fisher Scientific, Waltham, MA), 10 ng/ml recombinant human epidermal growth factor (rhEGF; Peprotech, Rocky Hill, NJ), and 50 nM hydrocortisone (HMEC‐1 medium; Sigma‐Aldrich, St. Louis, MO) up to passage 30. Where indicated, cells were stimulated with 0.5 µg/ml doxycycline (Sigma‐Aldrich) or with 5 ng/ml transforming growth factor‐β1 (TGF‐β1; Peprotech). For the study of intracellular regulatory pathways of EndMT, ECs were stimulated with doxycycline for 24 hr in the complete HMEC‐1 medium. EC stimulated with doxycycline in scratch wound migration and angiogenic sprouting assays were cultured in HMEC‐1 medium containing 0.2% FCS supplemented with doxycycline for 96 hr before the assays, as well as during the assays. For the study on EndMT markers, ECs were stimulated up to 7 days with doxycycline in HMEC‐1 medium containing 0.2% FCS.

### 2.2 Lentiviral knockdown and overexpression

Short hairpin‐mediated LOXL2 knockdown (shLOXL2) ECs and shRNA control (shCtrl) ECs were generated as described previously (de Jong et al., 2016). For the generation of a stable tet‐inducible LOXL2 overexpression EC line (Ind LOXL2), the LOXL2 open reading frame (ORF) from pCMV6‐XL5 LOXL2 (Origene, Rockville, MD) were flanked with attB1 and attB2 sites using the following PAGE‐purified oligonucleotides in a Phusion polymerase (New England Biolabs, Ipswitch, MA) PCR reaction: 5′‐GGGGACAAGTTTGTACAAAAAAGCAGGCTTCACCATGGAGAGGCCTCTGTGCTCCAC‐3′ and 5′‐GGGGACCACTTTGTACAAGAAAGCTGGGTTTTACTGCGGGGACAGCTGGTTGTT‐3′. The ORF was then recombined into pDONR221 using Gateway BP Clonase II Enzyme mix (Thermo Fisher Scientific). The LOXL2 ORF was subsequently recombined into pInducer20 (Meerbrey, Hu, & Kessler, 2011) using Gateway LR Clonase II Enzyme mix (Thermo Fisher Scientific). The catalytically inactive tet‐inducible LOXL2 H626/628Q EC line (Ind LOXL2 H626/628Q) was generated from pInducer20‐LOXL2 by site‐directed mutagenesis using the QuikChange II XL Site‐Directed Mutagenesis Kit (Agilent Technologies, Santa Clara, CA) with the following oligonucleotides: 5′‐CTCCATGCTGTGGTATTGCCTTTGACAGTCGTGCCAG‐3′ and 5′‐CTGGCACGACTGTCAAAGGCAATACCACAGCATGGAG‐3′. All generated constructs were fully sequenced to rule out undesirable mutations. Generation of lentiviral stocks and subsequent transfection of HMEC‐1 cells was performed as described previously (de Jong et al., 2016). Starting 24 hr after lentiviral transduction, HMEC‐1 cells were cultured and expanded in the presence of 2.5 µg/ml puromycin (pLKO.1 constructs) or 800 µg/ml G418 (pInducer20 constructs) for at least 1 week, and maintained in the presence of 2.5 µg/ml puromycin or 400 µg/ml G418 throughout further expansion. ECs were used up to maximum 10 passages to avoid loss of lentiviral constructs. Furthermore, cells of the same passage numbers, as well as passage numbers since lentiviral transduction, were used within experiments to correct for potential effects of cellular passage number and construct expression.

### 2.3 Immunoblotting

Cell samples were resuspended in RIPA buffer (Santa Cruz Biotechnology, Dallas, TX), and protein concentrations were determined by BCA protein assay (Thermo Fisher Scientific). Equal protein amounts were subjected to sodium dodecyl sulfate polyacrylamide gel electrophoresis (SDS‐PAGE) using NuPAGE 4–12% Bis‐Tris gradient gels or 12% Bis‐Tris gels (Thermo Fisher Scientific) alongside a prestained EZ‐Run protein ladder (Thermo Fisher Scientific), and subsequently transferred to polyvinylidene difluoride (PVDF) membranes (Thermo Fisher Scientific). Depending on the antibody, PVDF membranes were either blocked in 5% BSA (Roche, Basel, Switzerland) or 5% low‐fat dry milk powder (Campina, Woerden, The Netherlands) in tris‐buffered saline (TBS) with 0.1% Tween‐20 (TBST) for 1 hr at room temperature. PVDF membranes were incubated with primary antibodies in their respective blocking buffers overnight at 4°C. PVDF membranes were then washed in TBST, and incubated with horseradish peroxidase (HRP)‐conjugated secondary antibodies in 5% low‐fat dry milk powder in TBST for 1 hr at room temperature. PVDF membranes were subsequently washed in TBS and proteins were visualized using Chemiluminescent Peroxidase Substrate (Sigma‐Aldrich) and imaged on the Molecular Image ChemiDoc XRS system (Bio‐Rad, Hercules, CA). After imaging, PVDF membranes were stripped from antibodies using ReBlot Plus Mild Antibody Stripping Solution (Millipore, Billerica, MA) for 20 min at room temperature, blocked in 5% low‐fat dry milk powder in TBST, and reprobed for glyceraldehyde 3‐phosphate dehydrogenase (GAPDH) as a loading control. The primary antibodies used were LOXL2 (cat. no. AF2639; R&D Systems, Minneapolis, MN), GAPDH (cat. no. 2118; Cell Signaling Technology, Danvers, MA), α‐SMA (cat. no. TA310169; Origene), PECAM‐1 (cat. no. sc‐1505; Santa Cruz Biotechnology), VE‐cadherin (cat. no. TA804746; Origene), Snail (cat. no. TA500366; Origene), Fibronectin/HRP (cat. no. P0246; Dako, Glostrup, Denmark), FAK (cat. no. 13009; Cell Signaling Technology), p‐FAK Y397 (cat. no. 8556; Cell Signaling Technology), PKB/Akt (cat. no. 9272; Cell Signaling Technology), and p‐PKB/Akt S473 (cat. no. 4060; Cell Signaling Technology). Secondary antibodies used were HRP‐conjugated swine anti‐rabbit, rabbit anti‐mouse, and rabbit anti‐goat (all from Dako). Statistical analysis was performed using a one‐tail Student’s *t* test, after densitometric analysis using the ImageJ software (Laboratory for Optical and Computational Instrumentation, University of Wisconsin at Madison, WI).

### 2.4 Quantitative polymerase chain reaction (qPCR)

Total RNA was isolated from cells using TRIzol isolation (Thermo Fisher Scientific), according to the manufacturer’s protocol, followed by cDNA synthesis using the iScript cDNA Synthesis Kit (Bio‐Rad). qPCR was performed using IQ Sybr Green Super Mix (Bio‐Rad) in a CFX96 Real‐Time PCR Detection System (Bio‐Rad). Primer sequences were taken from the PrimerBank PCR primer public resource (Wang, 2003) and synthesized by Sigma‐Aldrich. The following primers were used: Ribosomal protein, large, p0 (RPLP0) (5′‐TCGACAATGGCAGCATCTAC‐3′, 5′‐ATCCGTCTCCACAGACAAGG‐3′), LOXL2 (5′‐GGGTGGAGGTGTACTATGATGG‐3′, 5′‐CTTGCCGTAGGAGGAGCTG‐3′), α‐SMA (5′‐CAGGGCTGTTTTCCCATCCAT‐3′, 5′‐ACGTAGCTGTCTTTTTGTCCC‐3′), calponin 1 (5′‐ATGTCCTCTGCTCACTTCAACC‐3′, 5′‐CCCCCTCGATCCACTCTCT‐3′), PECAM‐1 (5′‐AACAGTGTTGACATGAAGAGCC‐3′, 5′‐TGTAAAACAGCACGTCATCCTT‐3′), VE‐cadherin (5′‐TTGGAACCAGATGCACATTGAT‐3′, 5′‐TCTTGCGACTCACGCTTGAC‐3′), and fibronectin (5′‐GAAGCTCTCTCTCAGACAACCA‐3′, 5′‐GCCCACGGTAACAACCTCTT‐3′). Cycle threshold (*C*_t)_ values were normalized per experiment and per gene. ΔΔ*C*_t_ was calculated using housekeeping gene RPLP0. Statistical analysis was performed using a two‐tail Student’s *t* test.

### 2.5 Scratch migration assay

HMEC‐1 cells were plated in 24‐well plates, in a density that ensured full confluency during the assay. EC migration was measured by making a single, straight scratch in the cell monolayer, using a 200 µl pipet tip. Wells were then washed once with phosphate‐buffered saline (PBS), and 0.5 ml MCDB 131 with 100 U/ml penicillin and 100 µg/ml streptomycin (basal HMEC‐1 medium) was added to each well. Cells were incubated at 37°C for 6 hr. Images were recorded at *t* = 0 and *t* = 6 hr at the same location, and scratch closure was determined by measuring the scratch surface at both time points using the ImageJ software. Statistical analysis was performed using a two‐tail Student’s *t* test, or a two‐way analysis of variance (ANOVA) with Tukey’s multiple comparisons post hoc analysis to compare multiple groups.

### 2.6 Angiogenic sprouting assay

Angiogenic sprouting assays were performed as described in Balkom (2013); sprouting was assessed by seeding HMEC‐1 cells onto Cytodex 3 microcarrier beads (Sigma‐Aldrich), and embedding them in a mixture of basal HMEC‐1 medium, complete HMEC‐1 medium, and growth factor reduced Matrigel (Becton Dickinson, Franklin Lakes, NJ) in a 1:1:4 ratio, supplemented with doxycycline or a vehicle control where indicated, in a 100 µl volume in 48‐well plate wells. After the gels had solidified, 0.5 ml basal HMEC‐1 medium was added on top of the gels, and images were taken after a 72 hr incubation at 37°C, 5% CO_2_. Sprout lengths were measured using the ImageJ software. Ind LOXL2 EC and WT EC were stimulated with doxycycline or a vehicle control in HMEC‐1 medium with 0.2% FCS supplemented for 96 hr before the assay. Statistical analysis was performed using a two‐tail Student’s *t* test, or a two‐way ANOVA with Tukey’s multiple comparisons post hoc analysis to compare multiple groups.

### 2.7 Statistics

Data were normalized to means of each experiment with the reference condition set as 1. All statistical analyses were performed using Graphpad Prism 6.05. All values are expressed as the mean ± standard deviation (*SD*). Differences were considered statistically significant at *p* < 0.05.

## 3 RESULTS

### 3.1 LOXL2 regulates migration and angiogenic sprouting in endothelial cells

**Figure 1:**
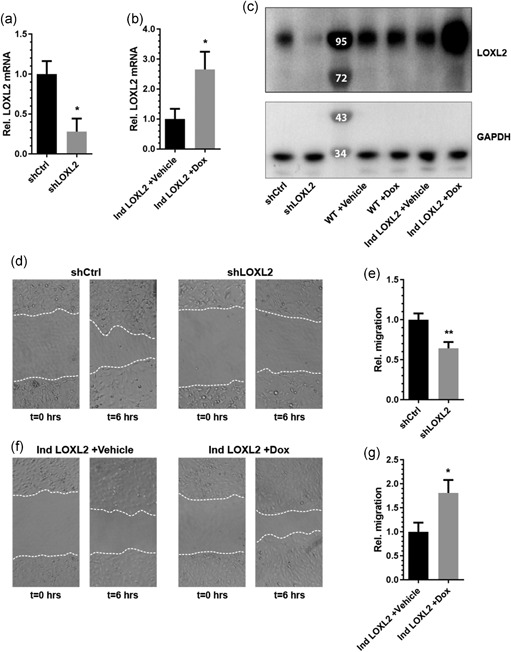
LOXL2 regulates migration in EC. (a) LOXL2 mRNA expression is decreased in shLOXL2 expressing EC compared to shCtrl expressing EC (*n* = 4 + *SD*, Student’s *t* test). (b) Doxycycline‐induced overexpression of LOXL2 mRNA is confirmed by stimulating EC expressing pInducer20‐LOXL2 (Ind LOXL2) with PBS (Vehicle) or doxycycline (Dox) for 24 hr. (c) Western blot analysis demonstrating reduced LOXL2 protein expression in shLOXL2 expressing EC, and increased LOXL2 protein expression when stimulating pInducer20‐LOXL2 infected EC (Ind LOXL2), but not wildtype EC (WT), with doxycycline, using GAPDH as a loading control. (d) Representative pictures of a migration scratch assay using shCtrl and shLOXL2 expressing EC at *t* = 0 and *t* = 6 hr. (e) Quantification of shCtrl and shLOXL2 expressing endothelial cell migration over 6 hr (*n* = 5 + *SD*, Student’s *t* test). (f) Representative pictures of a migration scratch assay when overexpressing LOXL2 in EC at *t* = 0 and *t* = 6 hr. (g) Quantification of LOXL2 overexpressing endothelial cell migration over 6 hr (*n* = 3 + *SD*, Student’s *t* test); *p < 0.05 and **p < 0.01. EC: endothelial cells; GAPDH: glyceraldehyde 3‐phosphate dehydrogenase; LOXL2: lysyl oxidase‐like 2; mRNA: messenger RNA; PBS: phosphate‐buffered saline; shCtrl: shRNA control; shLOXL2: short hairpin‐mediated LOXL2 knockdown

To verify the role of LOXL2 in angiogenesis, a LOXL2 knockdown cell line was generated by lentiviral transfection of HMEC‐1 with LOXL2‐targeting short hairpin RNAs (shLOXL2; as described by de Jong et al., 2016). To study the effects of LOXL2 overexpression, we generated a tet‐inducible LOXL2 overexpressing EC cell line (Ind LOXL2). For the LOXL2 knockdown cell line, LOXL2 mRNA and protein expression knockdown were confirmed by qPCR and immunoblot, respectively (Figure 1a,c). In the inducible overexpression cell line, increased LOXL2 mRNA and protein levels after doxycycline stimulation were confirmed by qPCR and immunoblot analysis (Figure 1b,c). LOXL2 knockdown strongly reduced EC migration, as assessed by a scratch wound assay (Figure 1d,e) whereas doxycyclin‐induced LOXL2 overexpression induced a substantial and significant increase in EC migration (Figure 1f,g). As a control, stimulation of wildtype EC with doxycycline showed no significant effect on endothelial migration (Supporting Information Figure S1a).

**Figure 2:**
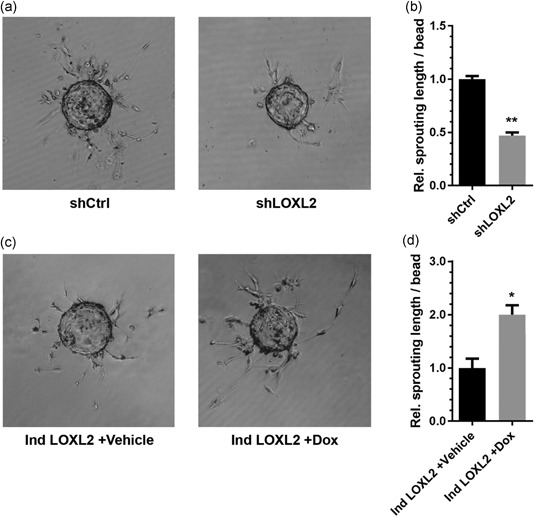
LOXL2 regulates angiogenic sprouting in EC. (a) Representative pictures of an angiogenic sprouting assay using shCtrl and shLOXL2 expressing EC at *t* = 72 hr. (b) Quantification of sprout length in a 72 hr angiogenic sprouting assay using shCtrl and shLOXL2 expressing EC (*n* = 4 + *SD*, Student’s *t* test). (c) Representative pictures of an angiogenic sprouting assay in pInducer20‐LOXL2 (Ind LOXL2) expressing EC stimulated with PBS (Vehicle) or doxycycline (Dox) at *t* = 72 hr. (d) LOXL2 overexpression increases sprouting length in an angiogenic sprouting assay (*n* = 4 + *SD*, Student’s *t* test); *p < 0.05 and **p < 0.01. EC: endothelial cells; LOXL2: lysyl oxidase‐like 2; mRNA: messenger RNA; PBS: phosphate‐buffered saline; shCtrl: shRNA control; shLOXL2: short hairpin‐mediated LOXL2 knockdown

Additionally, LOXL2 knockdown dramatically reduced sprouting length in an in vitro angiogenic sprouting assay (Figure 2a,b) in contrast to LOXL2 overexpression, which significantly increased angiogenic sprouting in EC (Figure 2c,d). LOXL2 knockdown reduced the number of angiogenic sprouts per bead, but not the average sprouting length (Supporting Information Figure S2a,b), whereas overexpression of LOXL2 resulted in a significant increase of the number of angiogenic sprouts per bead, as well as the average sprouting length (Supporting Information Figure S2c,d). In a control experiment, stimulation of wildtype EC with doxycycline showed no significant effect on angiogenic sprouting (Supporting Information Figure S1b).

### 3.2 LOXL2 induces angiogenesis independent of its enzymatic activity

**Figure 3:**
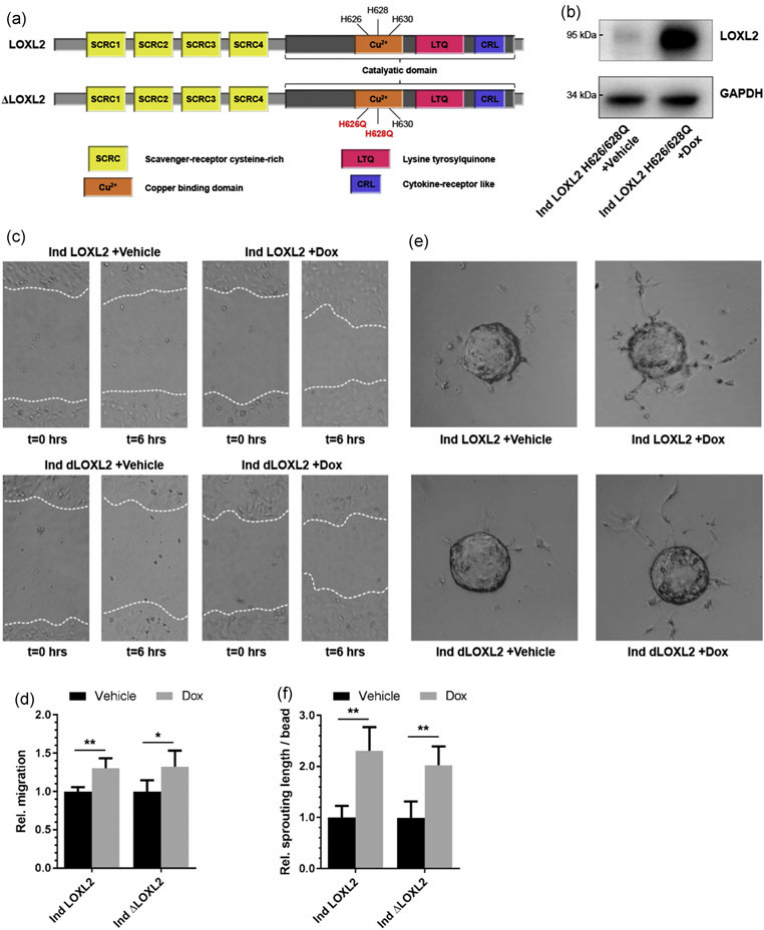
LOXL2‐induced increase in migration and sprouting is independent of enzymatic activity. (a) A schematic representation of LOXL2 (upper panel), and the catalytically inactive H26Q‐H628Q‐LOXL2 mutant (LOXL2 H626/628Q). (b) Western blot analysis demonstrating the inducible overexpression of an H26Q‐H628Q‐LOXL2 mutant in EC by stimulation with doxycycline (Dox) for 24 hr compared to PBS (Vehicle). (c) Representative pictures of a migration scratch assay when overexpressing wildtype LOXL2 (Ind LOXL2) and the H26Q‐H628Q‐LOXL2 mutant (Ind dLOXL2) in EC at *t* = 0 and *t* = 6 hr. (d) Overexpression of either wildtype LOXL2 or LOXL2 H626/628Q in EC results in comparable increased migration in scratch migration assays (*n* = 4 + *SD*, Student’s *t* test). (e) Representative pictures of an angiogenic sprouting assay in inducible wildtype LOXL2 or LOXL2 H626/628Q expressing EC stimulated with PBS (Vehicle) or doxycycline (Dox) at *t* = 72 hr. (f) Overexpression of either wildtype LOXL2 or LOXL2 H626/628Q in EC results in a comparable increase in sprouting length in angiogenic sprouting assays (*n* = 4 + *SD*, Student’s *t* test); *p < 0.05 and **p < 0.01. EC: endothelial cells; GAPDH: glyceraldehyde 3‐phosphate dehydrogenase; LOXL2: lysyl oxidase‐like 2; PBS: phosphate‐buffered saline [Color figure can be viewed at wileyonlinelibrary.com]

To determine whether the positive effect of LOXL2 overexpression on EC migration and sprouting is due to enzymatic crosslinking of collagens and elastins by LOXL2, a catalytically inactive mutant of LOXL2 was generated by introducing two‐point mutations, replacing the histidines at positions H626 and H628 with glutamine residues (Ind LOXL2 H626/628Q; Figure 3a). These mutations have been reported to completely abolish the oxidative deamination activity of LOXL2, the process through which lysyl oxidases crosslink collagens and elastins, and not affect the induction of EMT in epithelial cells (Cuevas, Moreno‐Bueno, & Canesin, 2014). Inducible overexpression of LOXL2 H626/628Q in EC was confirmed by immunoblot analysis (Figure 3b). Interestingly, overexpression of this catalytically inactive LOXL2 mutant resulted in a similar increase in EC migration as seen in EC with wildtype LOXL2 overexpression (Figure 3c,d). Additionally, a similar increase in angiogenic sprouting as a result of overexpression of wildtype LOXL2 and LOXL2 H626/628Q was observed (Figure 3e,f). These data show that the effects of LOXL2 on EC migration and sprouting do not depend on LOXL2‐mediated enzymatic crosslinking of ECM components, but rather point toward a mechanism involving LOXL2‐mediated intracellular signaling.

### 3.3 LOXL2 is required but not sufficient to induce EndMT

**Figure 4:**
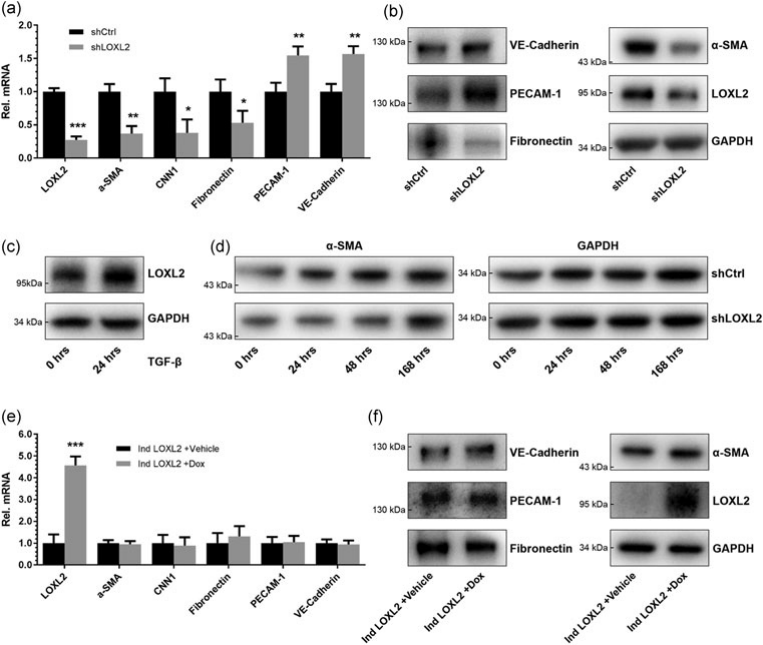
LOXL2 knockdown in EC reduces EndMT, but LOXL2 overexpression does not induce EndMT. (a) LOXL2 knockdown in EC reduces mRNA expression of mesenchymal markers α‐SMA, calponin 1, and fibronectin, and increases mRNA expression of endothelial markers PECAM‐1 and VE‐cadherin (*n* = 3, + *SD*, Student’s *t* test). (b) LOXL2 knockdown in EC increases protein expression of endothelial markers PECAM‐1 and VE‐cadherin, and reduces protein expression of mesenchymal markers α‐SMA and fibronectin. (c) The LOXL2 expression is increased in EC after stimulation with TGF‐β for 24 hr. (d) LOXL2 knockdown results in delayed TGF‐β‐mediated upregulation of α‐SMA in EC. (e) LOXL2 overexpression does not affect mRNA expression of mesenchymal markers α‐SMA, calponin 1, and fibronectin, or expression of endothelial markers PECAM‐1 and VE‐cadherin (*n* = 3, + *SD*, Student’s *t* test). (f) LOXL2 overexpression does not affect protein expression of endothelial markers PECAM‐1 and VE‐cadherin, or expression of mesenchymal markers α‐SMA and fibronectin; *p < 0.05, **p < 0.01, and ***p < 0.001. EC: endothelial cells; EndMT: endothelial‐to‐mesenchymal transition; GAPDH: glyceraldehyde 3‐phosphate dehydrogenase; LOXL2: lysyl oxidase‐like 2; mRNA: messenger RNA; PBS: phosphate‐buffered saline; PECAM‐1: platelet endothelial cell adhesion molecule 1; shCtrl: shRNA control; α‐SMA: α‐smooth muscle actin; shLOXL2: short hairpin‐mediated LOXL2 knockdown; TGF‐β: transforming growth factor‐β; VE‐cadherin: vascular endothelial cadherin

Given the known stimulatory effects of EndMT on angiogenesis (Muylaert et al., 2015; van Meeteren & ten Dijke, 2012) and the reported dominant role of LOXL2 in the regulation of EMT (Peinado, 2005; Peinado et al., 2005), we hypothesized that the observed results may be explained by a similar regulatory function of LOXL2 in EndMT. To assess whether LOXL2 indeed plays a role in the regulation of EndMT, shLOXL2 EC were analyzed for expression of the endothelial markers VE‐cadherin and PECAM‐1, and mesenchymal markers α‐SMA, calponin 1, and fibronectin. qPCR analysis revealed an increased expression of endothelial markers and a decreased expression of mesenchymal markers (Figure 4a). Upregulation of VE‐cadherin and PECAM‐1, as well as downregulation of α‐SMA and fibronectin, were also confirmed by immunoblot analysis (Figure 4b). These observed changes indicate a balance toward a more endothelial phenotype, in line with a decrease in EndMT. To determine whether LOXL2 plays a role in the TGF‐β‐mediated regulation of EndMT, LOXL2 expression was measured after stimulation of wildtype EC with TGF‐β for 24 hr. Indeed, a substantial increase of LOXL2 expression was observed (Figure 4c). To further assess the role of LOXL2 in EndMT, we exposed shLOXL2 EC to TGF‐β for 7 days (168 hr) to induce EndMT, and measured expression of EndMT marker α‐SMA (Figure 4d). Immunoblot analysis showed a significantly lower expression of α‐SMA in shLOXL2 EC compared with control EC (shCtrl) after 24, 48, and 96 hr of TGF‐β stimulation (*p* = 0.04, 0.02, and 0.04, respectively; *n* = 4), but not after 168 hr, indicating a delayed, but not blocked, response to TGF‐β. This demonstrates a LOXL2‐dependent regulation of EndMT. To investigate whether LOXL2 upregulation is sufficient to induce EndMT, Ind LOXL2 EC were stimulated with doxycycline for 7 days to establish continuous LOXL2 overexpression. Interestingly, no significant changes in EndMT marker expression could be observed by both qPCR (Figure 4e) and immunoblot analysis (Figure 4f). As a control, stimulation of wildtype EC with doxycycline showed no significant effect on EndMT marker mRNA levels (Supporting Information Figure S1c). These data show that LOXL2 plays a regulatory role in EndMT, but overexpression alone is not sufficient to induce EndMT.

### 3.4 LOXL2 stimulates PKB/Akt and FAK signaling

**Figure 5:**
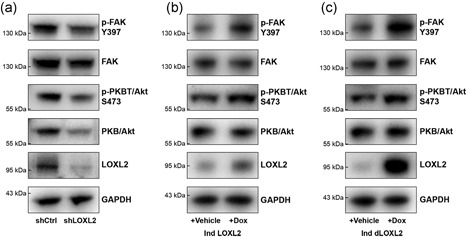
LOXL2 regulates EMT–EndMT‐associated signaling pathways PKB/Akt and FAK. (a) Knockdown of LOXL2 in EC reduces FAK Y397 phosphorylation, and reduces PKB/Akt expression and phosphorylation. (b) Overexpression of LOXL2 in EC increases FAK Y397 phosphorylation and PKB/Akt S473. (c) Overexpression of the catalytically inactive H26Q‐H628Q‐LOXL2 mutant increases FAK Y397 phosphorylation and PKB/Akt S473. EC: endothelial cells; EndMT: endothelial‐to‐mesenchymal transition; EMT: epithelial‐to‐mesenchymal transition; GAPDH: glyceraldehyde 3‐phosphate dehydrogenase; FAK: focal adhesion kinase; LOXL2: lysyl oxidase‐like 2; PKB: protein kinase B

LOXL2 thus appears crucial but not sufficient for the induction of EndMT. To better understand the stimulating role of LOXL2 in EC sprouting and migration, we further investigated pathways involved in the regulation of EndMT and EMT. Given the reported roles of protein kinase B (PKB)/Akt and focal adhesion kinase (FAK) signaling in angiogenesis, as well as the regulation of EMT and EndMT (Deng, Yang, & Liu, 2010; Larue & Bellacosa, 2005; Meadows, Iyer, & Stevens, 2009; Shen, Park, & Alcaraz, 2005), we studied the effect of LOXL2 knockdown and overexpression on these regulatory pathways. We found that knockdown of LOXL2 resulted in decreased PKB/Akt and FAK signaling: Phosphorylation of FAK was decreased, and expression of PKB/Akt—as a result presence of phosphorylated PKB/Akt—as well was substantially decreased (Figure 5a). Concordantly, overexpression of LOXL2 for 24 hr resulted in an increase in phosphorylation of PKB/Akt and FAK (Figure 5b). Similarly, overexpression of the catalytically inactive LOXL2 H626/628Q resulted in increased phosphorylation of PKB/Akt and FAK as well, demonstrating that activation of these signaling pathways is independent of the enzymatic activity of LOXL2 (Figure 5c). Unlike in EMT (Peinado, 2005; Peinado et al., 2005), knockdown or overexpression of LOXL2 did not affect the expression of Snail (Supporting Information Figure S3).

## 4 DISCUSSION

Here we demonstrate that LOXL2 is a regulator of EndMT, and through this process plays a regulatory role in angiogenesis. By studying the effects of LOXL2 knockdown, overexpression, and overexpression of a catalytically inactive mutant of LOXL2 in EC, we determined the respective roles of LOXL2 enzymatic activity and its intracellular signaling in the regulation of angiogenesis. While a role for LOXL2 in angiogenesis and embryonic development through lysyl oxidase‐mediated ECM remodeling has previously been reported, we show for the first time that LOXL2 is also involved in additional intracellular signaling pathways in EC. These findings complement the previously reported effects of LOXL2‐mediated ECM crosslinking activity on angiogenesis (Bignon et al., 2011).

LOXL2 has previously been reported to regulate EMT in epithelial cells through various intracellular pathways, including suppression of E‐cadherin expression by lysine residue deamination in histone H3 and through regulation of Snail (Herranz et al., 2016; Peinado et al., 2005). Given the reported role of LOXL2 in the regulation of EMT, we hypothesized that LOXL2 plays a similar intracellular role in the regulation of EndMT. Indeed, LOXL2 knockdown in EC resulted in decreased migration, angiogenic sprouting, expression of mesenchymal markers, and an increase in expression of endothelial markers. Unlike in EMT (Peinado, 2005; Peinado et al., 2005) however, LOXL2 overexpression was not sufficient to induce EndMT. An intriguing result, as many of the regulatory pathways of EndMT and EMT overlap, including Snail, PKB/Akt, and zinc finger E‐box‐binding homeobox 1 (ZEB1) signaling (Aigner, Dampier, & Descovich, 2007; Kokudo, Suzuki, & Yoshimatsu, 2008; Larue & Bellacosa, 2005; Meadows et al., 2009; Medici et al., 2011; Peinado, 2005). However, differences in regulation of EndMT and EMT have been reported. For example, whereas Snail overexpression is sufficient to induce EMT (Cano, Perez‐Moreno, & Rodrigo, 2000), Snail overexpression alone is not sufficient to induce EndMT; alongside overexpression of Snail, additional downregulation of glycogen synthase kinase‐3 beta (GSK‐3β) activity, which is involved in the regulation of angiogenesis and EC morphogenesis (Shin, Wolgamott, & Yoon, 2012) is required (Medici et al., 2011). Furthermore, whereas hypoxia induces EMT through hypoxia‐inducible transcription factor‐1α (HIF‐1α)‐mediated upregulation of LOXL2 (Schietke, Warnecke, & Wacker, 2010), hypoxia inhibits EndMT through vascular endothelial growth factor (VEGF) signaling (Dor, Camenisch, & Itin, 2001).

These findings indicate that EndMT, as compared to EMT, is controlled by an additional layer of regulatory pathways, which might be in place to protect EC function to maintain the integrity of the circulatory system, as inappropriate EndMT can result in fibrosis and atherosclerosis (Chen, Qin, & Baeyens, 2015; Elliott, Gurtu, & McCollum, 2014; Zeisberg, Tarnavski, & Zeisberg, 2007).

Our data indicate that the effects of LOXL2 overexpression on migration and sprouting are due to activation of pathways that are involved in the regulation of EndMT and EMT, but are not sufficient to induce full EndMT in current conditions. Indeed, we find that LOXL2 expression regulates FAK signaling, which has been implicated in the regulation of EMT (Deng et al., 2010), and PKB/Akt signaling, which has been implicated in the regulation of both EndMT and EMT (Larue & Bellacosa, 2005; Meadows et al., 2009). Additionally, we find that LOXL2 expression is increased by TGF‐β stimulation in EC. TGF‐β‐mediated regulation of LOXL2 had been reported in hepatocellular carcinoma (Wong, Tse, & Huang, 2014) but had not yet been described in endothelial cells. Importantly, LOXL2 knockdown also resulted in delayed TGF‐β‐mediated EndMT, confirming its crucial role in the regulation of EndMT.

In conclusion, we demonstrate a regulatory role for LOXL2 in adult endothelial cells during angiogenesis and EndMT, through intracellular signaling independent of its ECM remodeling activity. These results shed an additional light on the crucial physiological function of LOXL2 in angiogenesis.

## AUTHOR CONTRIBUTIONS

O. G. d. J. designed the study, performed experiments, and wrote the manuscript. L. M. v. d. W. and F. R. W. K. performed experiments. B. W. M. v. B. designed the study, supervised the project and contributed to the preparation of the manuscript. M. C. V. supervised the project and contributed to the preparation of the manuscript. All authors reviewed and approved the final version of the manuscript.

## CONFLICTS OF INTEREST

The authors declare that there are no conflicts of interest.

## Supporting information

Supporting information

Supporting information

Supporting information

Supporting information

## References

[jcp27695-bib-0001] Ades, E. W. , Candal, F. J. , Swerlick, R. A. , George, V. G. , Summers, S. , Bosse, D. C. , & Lawley, T. J. (1992). HMEC‐1: Establishment of an immortalized human microvascular endothelial cell line. Journal of Investigative Dermatology, 99, 683–690. 1361507 10.1111/1523-1747.ep12613748

[jcp27695-bib-0002] Ahn, S. G. , Dong, S. M. , Oshima, A. , Kim, W. H. , Lee, H. M. , Lee, S. A. , … Green, J. E. (2013). LOXL2 expression is associated with invasiveness and negatively influences survival in breast cancer patients. Breast Cancer Research and Treatment, 141, 89–99. 23933800 10.1007/s10549-013-2662-3 PMC6944271

[jcp27695-bib-0003] Aigner, K. , Dampier, B. , Descovich, L. , Mikula, M. , Sultan, A. , Schreiber, M. , … Eger, A. (2007). The transcription factor ZEB1 (deltaEF1) promotes tumour cell dedifferentiation by repressing master regulators of epithelial polarity. Oncogene, 26, 6979–6988. 17486063 10.1038/sj.onc.1210508 PMC2899859

[jcp27695-bib-0004] van Balkom, B. W. M. , de Jong, O. G. , Smits, M. , Brummelman, J. , den Ouden, K. , de Bree, P. M. , … Verhaar, M. C. (2013). Endothelial cells require miR‐214 to secrete exosomes that suppress senescence and induce angiogenesis in human and mouse endothelial cells. Blood, 121, 3997–4006. 23532734 10.1182/blood-2013-02-478925

[jcp27695-bib-0005] Bignon, M. , Pichol‐Thievend, C. , Hardouin, J. , Malbouyres, M. , Brechot, N. , Nasciutti, L. , … Germain, S. (2011). Lysyl oxidase‐like protein‐2 regulates sprouting angiogenesis and type IV collagen assembly in the endothelial basement membrane. Blood, 118, 3979–3989. 21835952 10.1182/blood-2010-10-313296

[jcp27695-bib-0006] Cano, A. , Pérez‐Moreno, M. A. , Rodrigo, I. , Locascio, A. , Blanco, M. J. , del Barrio, M. G. , … Nieto, M. A. (2000). The transcription factor snail controls epithelial‐mesenchymal transitions by repressing E‐cadherin expression. Nature Cell Biology, 2, 76–83. 10655586 10.1038/35000025

[jcp27695-bib-0007] Chang, J. , Nicolau, M. M. , Cox, T. R. , Wetterskog, D. , Martens, J. W. , E Barker, H. , & Erler, J. T. (2013). LOXL2 induces aberrant acinar morphogenesis via ErbB2 signaling. Breast Cancer Research, 15, R67. 23971878 10.1186/bcr3461 PMC3978831

[jcp27695-bib-0008] Chen, P. Y. , Qin, L. , Baeyens, N. , Li, G. , Afolabi, T. , Budatha, M. , … Simons, M. (2015). Endothelial‐to‐mesenchymal transition drives atherosclerosis progression. Journal of Clinical Investigation, 125, 2015–4528. 10.1172/JCI82719 PMC4665771 26517696

[jcp27695-bib-0009] Cuevas, E. P. , Moreno‐Bueno, G. , Canesin, G. , Santos, V. , Portillo, F. , & Cano, A. (2014). LOXL2 catalytically inactive mutants mediate epithelial‐to‐mesenchymal transition. Biology Open, 3, 129–137. 24414204 10.1242/bio.20146841 PMC3925316

[jcp27695-bib-0010] Deng, B. , Yang, X. , Liu, J. , He, F. , Zhu, Z. , & Zhang, C. (2010). Focal adhesion kinase mediates TGF‐beta1‐induced renal tubular epithelial‐to‐mesenchymal transition in vitro. Molecular and Cellular Biochemistry, 340, 21–29. 20177740 10.1007/s11010-010-0396-7

[jcp27695-bib-0011] Dor, Y. , Camenisch, T. D. , Itin, A. , Fishman, G. I. , McDonald, J. A. , Carmeliet, P. , & Keshet, E. (2001). A novel role for VEGF in endocardial cushion formation and its potential contribution to congenital heart defects. Development, 128, 1531–1538. 11290292 10.1242/dev.128.9.1531

[jcp27695-bib-0012] Elliott, G. C. , Gurtu, R. , McCollum, C. , Newman, W. G. , & Wang, T. (2014). Foramen ovale closure is a process of endothelial‐to‐mesenchymal transition leading to fibrosis. PLOS One, 9, e107175. 25215881 10.1371/journal.pone.0107175 PMC4162597

[jcp27695-bib-0013] Herranz, N. , Dave, N. , Millanes‐Romero, A. , Pascual‐Reguant, L. , Morey, L. , Díaz, V. M. , … Peiró, S. (2016). Lysyl oxidase‐like 2 (LOXL2) oxidizes trimethylated lysine 4 in histone H3. FEBS Journal, 283, 4263–4273. 27735137 10.1111/febs.13922

[jcp27695-bib-0014] Iturbide, A. , Pascual‐Reguant, L. , Fargas, L. , Cebrià, J. P. , Alsina, B. , García de Herreros, A. , & Peiró, S. (2015). LOXL2 oxidizes methylated TAF10 and controls TFIID‐dependent genes during neural progenitor differentiation. Molecular Cell, 58, 755–766. 25959397 10.1016/j.molcel.2015.04.012

[jcp27695-bib-0015] de Jong, O. G. , van Balkom, B. W. M. , Gremmels, H. , & Verhaar, M. C. (2016). Exosomes from hypoxic endothelial cells have increased crosslinking activity through up‐regulation of lysyl oxidase‐like 2. Journal of Cellular and Molecular Medicine, 20, 342–350. 26612622 10.1111/jcmm.12730 PMC4727569

[jcp27695-bib-0016] de Jong, O. G. , Verhaar, M. C. , Chen, Y. , Vader, P. , Gremmels, H. , Posthuma, G. , … van Balkom, B. W. M. (2012). Cellular stress conditions are reflected in the protein and RNA content of endothelial cell‐derived exosomes. Journal of Extracellular Vesicles, 1, 1. 10.3402/jev.v1i0.18396 PMC3760650 24009886

[jcp27695-bib-0017] Kim, Y. M. , Kim, E. C. , & Kim, Y. (2011). The human lysyl oxidase‐like 2 protein functions as an amine oxidase toward collagen and elastin. Molecular Biology Reports, 38, 145–149. 20306300 10.1007/s11033-010-0088-0

[jcp27695-bib-0018] Kokudo, T. , Suzuki, Y. , Yoshimatsu, Y. , Yamazaki, T. , Watabe, T. , & Miyazono, K. (2008). Snail is required for TGFbeta‐induced endothelial‐mesenchymal transition of embryonic stem cell‐derived endothelial cells. Journal of Cell Science, 121, 3317–3324. 18796538 10.1242/jcs.028282

[jcp27695-bib-0019] Kovacic, J. C. , Mercader, N. , Torres, M. , Boehm, M. , & Fuster, V. (2012). Epithelial‐to‐mesenchymal and endothelial‐to‐mesenchymal transition: From cardiovascular development to disease. Circulation, 125, 1795–1808. 22492947 10.1161/CIRCULATIONAHA.111.040352 PMC3333843

[jcp27695-bib-0020] Larue, L. , & Bellacosa, A. (2005). Epithelial‐mesenchymal transition in development and cancer: Role of phosphatidylinositol 3′ kinase/AKT pathways. Oncogene, 24, 7443–7454. 16288291 10.1038/sj.onc.1209091

[jcp27695-bib-0021] Lin, F. , Wang, N. , & Zhang, T. C. (2012). The role of endothelial‐mesenchymal transition in development and pathological process. IUBMB Life, 64, 717–723. 22730243 10.1002/iub.1059

[jcp27695-bib-0022] Meadows, K. N. , Iyer, S. , Stevens, M. V. , Wang, D. , Shechter, S. , Perruzzi, C. , … Benjamin, L. E. (2009). Akt promotes endocardial‐mesenchyme transition. Journal of Angiogenesis Research, 1, 2. 19946410 10.1186/2040-2384-1-2 PMC2776235

[jcp27695-bib-0023] Medici, D. , Potenta, S. , & Kalluri, R. (2011). Transforming growth factor‐beta2 promotes Snail‐mediated endothelial‐mesenchymal transition through convergence of Smad‐dependent and Smad‐independent signalling. Biochemical Journal, 437, 515–520. 21585337 10.1042/BJ20101500 PMC4457510

[jcp27695-bib-0024] Meerbrey, K. L. , Hu, G. , Kessler, J. D. , Roarty, K. , Li, M. Z. , Fang, J. E. , … Elledge, S. J. (2011). The pINDUCER lentiviral toolkit for inducible RNA interference in vitro and in vivo. Proceedings of the National Academy of Sciences of the United States of America, 108, 3665–3670. 21307310 10.1073/pnas.1019736108 PMC3048138

[jcp27695-bib-0025] van Meeteren, L. A. , & ten Dijke, P. (2012). Regulation of endothelial cell plasticity by TGF‐beta. Cell and Tissue Research, 347, 177–186. 21866313 10.1007/s00441-011-1222-6 PMC3250609

[jcp27695-bib-0026] Muylaert, D. E. , de Jong, O. G. , Slaats, G. G. , Nieuweboer, F. E. , Fledderus, J. O. , Goumans, M. J. , … Verhaar, M. C. (2015). Environmental influences on endothelial to mesenchymal transition in developing implanted cardiovascular tissue‐engineered grafts. Tissue Engineering. Part B, Reviews, 22, 58–67. 10.1089/ten.TEB.2015.0167 26414174

[jcp27695-bib-0027] Peinado, H. , del Carmen Iglesias‐de la Cruz, M. , Olmeda, D. , Csiszar, K. , Fong, K. S. K. , Vega, S. , … Portillo, F. (2005). A molecular role for lysyl oxidase‐like 2 enzyme in snail regulation and tumor progression. EMBO Journal, 24, 3446–3458. 16096638 10.1038/sj.emboj.7600781 PMC1276164

[jcp27695-bib-0028] Peinado, H. , Portillo, F. , & Cano, A. (2005). Switching on‐off Snail: LOXL2 versus GSK3beta. Cell Cycle, 4, 1749–1752. 16294032 10.4161/cc.4.12.2224

[jcp27695-bib-0029] Schietke, R. , Warnecke, C. , Wacker, I. , Schödel, J. , Mole, D. R. , Campean, V. , … Wiesener, M. S. (2010). The lysyl oxidases LOX and LOXL2 are necessary and sufficient to repress E‐cadherin in hypoxia: Insights into cellular transformation processes mediated by HIF‐1. Journal of Biological Chemistry, 285, 6658–6669. 20026874 10.1074/jbc.M109.042424 PMC2825461

[jcp27695-bib-0030] Shen, T. L. , Park, A. Y. J. , Alcaraz, A. , Peng, X. , Jang, I. , Koni, P. , … Guan, J. L. (2005). Conditional knockout of focal adhesion kinase in endothelial cells reveals its role in angiogenesis and vascular development in late embryogenesis. Journal of Cell Biology, 169, 941–952. 15967814 10.1083/jcb.200411155 PMC2171636

[jcp27695-bib-0031] Shin, S. , Wolgamott, L. , & Yoon, S. O. (2012). Regulation of endothelial cell morphogenesis by the protein kinase D (PKD)/glycogen synthase kinase 3 (GSK3)beta pathway. American Journal of Physiology: Cell Physiology, 303, C743–C756. 22855295 10.1152/ajpcell.00442.2011

[jcp27695-bib-0032] Wang, X. A. (2003). PCR primer bank for quantitative gene expression analysis. Nucleic Acids Research, 31, 154e–154e. 10.1093/nar/gng154 PMC291882 14654707

[jcp27695-bib-0033] Welch‐Reardon, K. M. , Ehsan, S. M. , Wang, K. , Wu, N. , Newman, A. C. , Romero‐Lopez, M. , … Hughes, C. C. W. (2014). Angiogenic sprouting is regulated by endothelial cell expression of Slug. Journal of Cell Science, 127, 2017–2028. 24554431 10.1242/jcs.143420 PMC4004976

[jcp27695-bib-0034] Wong, C. C. L. , Tse, A. P. W. , Huang, Y. P. , Zhu, Y. T. , Chiu, D. K. C. , Lai, R. K. H. , … Ng, I. O. L. (2014). Lysyl oxidase‐like 2 is critical to tumor microenvironment and metastatic niche formation in hepatocellular carcinoma. Hepatology, 60, 1645–1658. 25048396 10.1002/hep.27320

[jcp27695-bib-0035] Zeisberg, E. M. , Tarnavski, O. , Zeisberg, M. , Dorfman, A. L. , McMullen, J. R. , Gustafsson, E. , … Kalluri, R. (2007). Endothelial‐to‐mesenchymal transition contributes to cardiac fibrosis. Nature Medicine (New York, NY, United States), 13, 952–961. 10.1038/nm1613 17660828

