# Supplementary figures and images for: Lysyl oxidase‐like 2 is a regulator of angiogenesis through modulation of endothelial‐to‐mesenchymal transition
Source: J Cell Physiol. 2018 Nov 1;234(7):10260–9. doi: 10.1002/jcp.27695 (PMC6587725; doi:10.1002/jcp.27695)

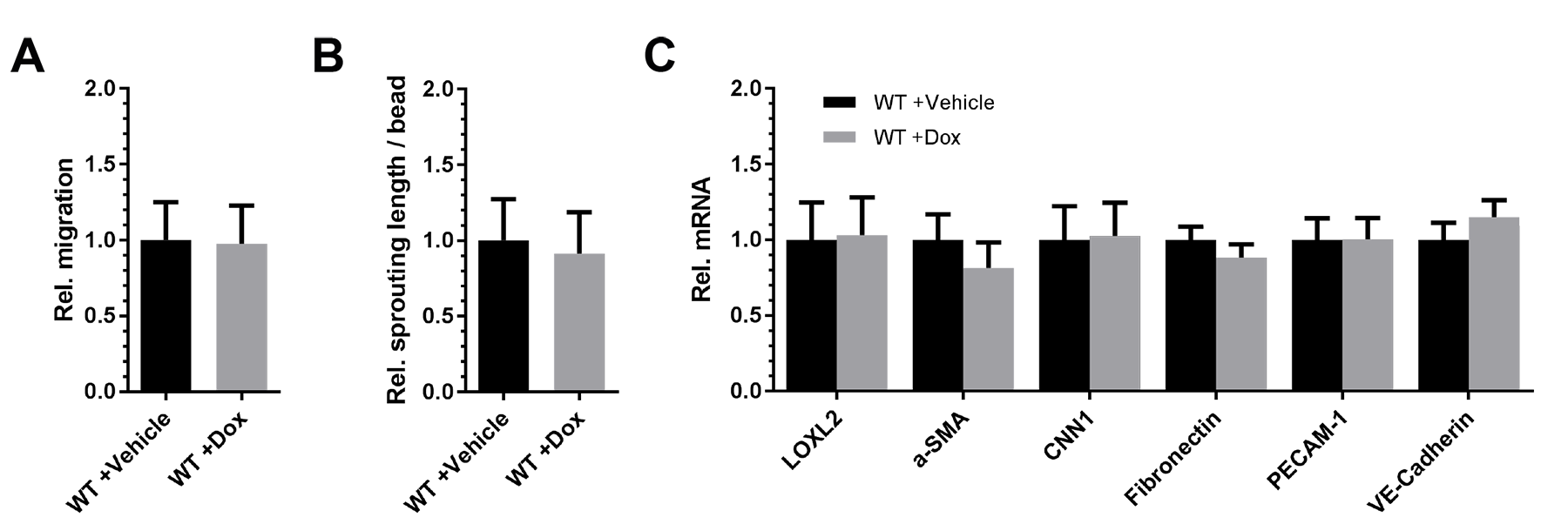

Supplement: Supplementary file 1 — Supporting information [file JCP-234-10260-s001.tif]

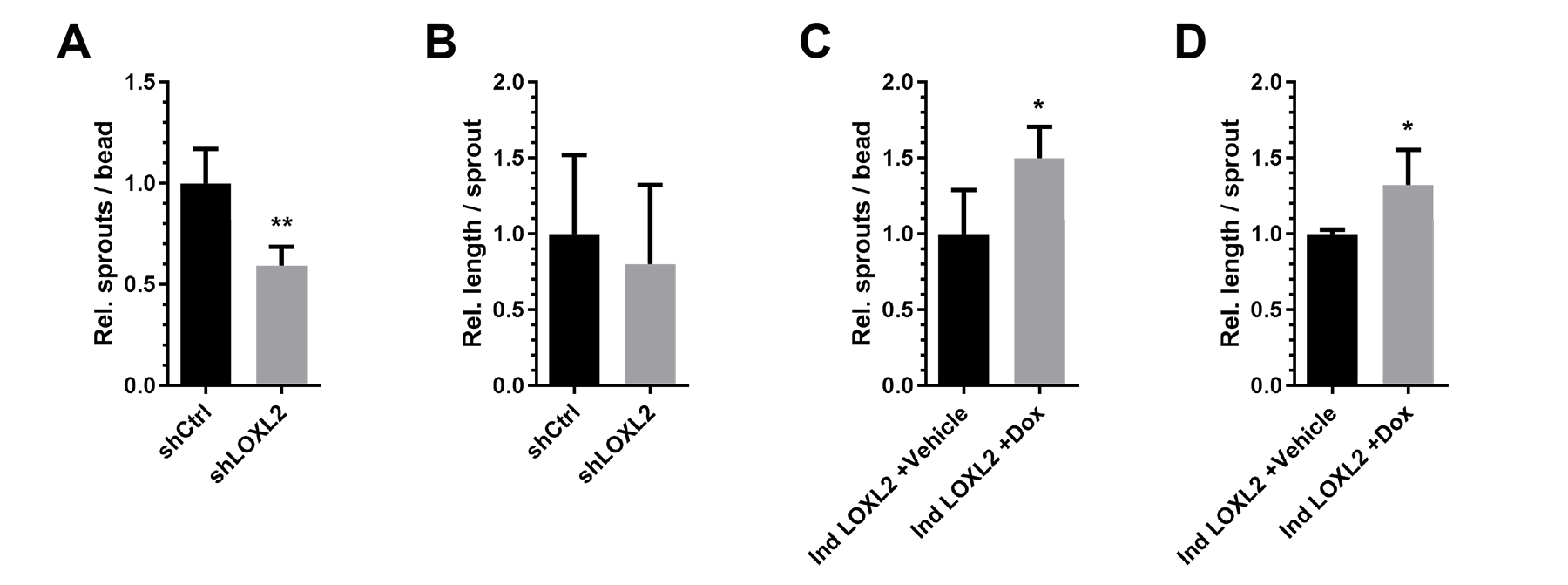

Supplement: Supplementary file 2 — Supporting information [file JCP-234-10260-s002.tif]

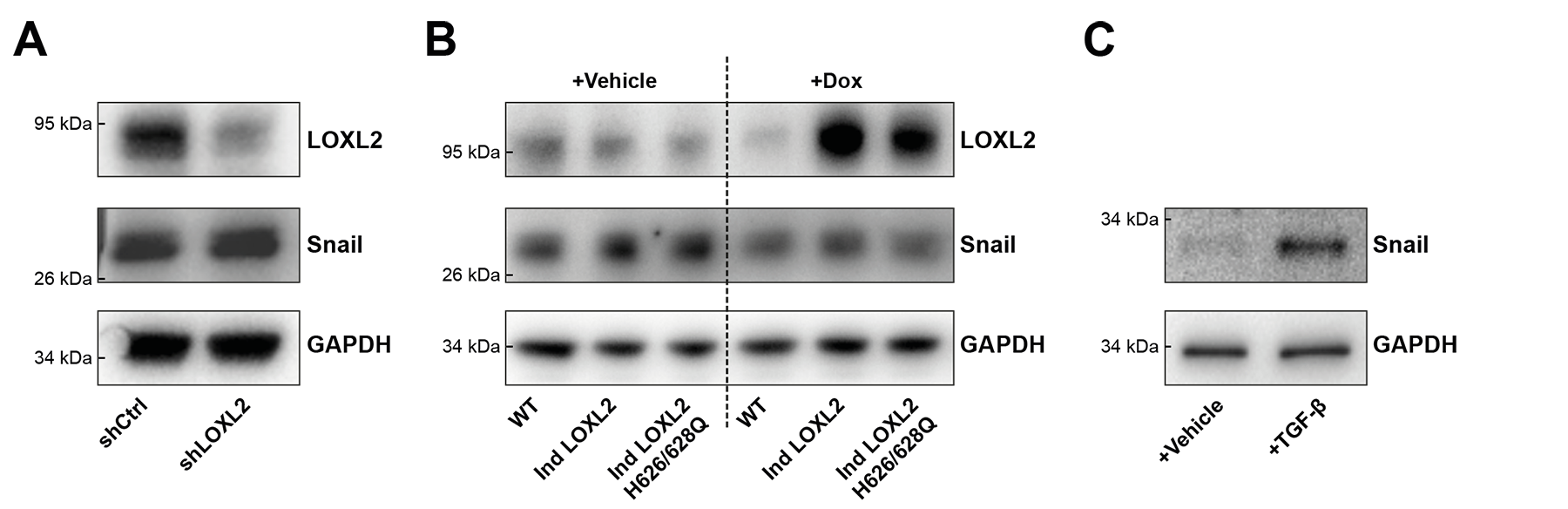

Supplement: Supplementary file 3 — Supporting information [file JCP-234-10260-s003.tif]
