# Supplementary material for: Lysyl oxidase‐like 2 is a regulator of angiogenesis through modulation of endothelial‐to‐mesenchymal transition
Source: J Cell Physiol. 2018 Nov 1;234(7):10260–9. doi: 10.1002/jcp.27695 (PMC6587725; doi:10.1002/jcp.27695)
Supplement: Supplementary file 4 — Supporting information [file JCP-234-10260-s004.docx]

**Figure S1. Doxycycline has no effect on migration, sprouting, and EndMT in wildtype EC.** (A) WT EC migration is not affected by doxycycline simulation in 6 hr scratch migration assays (n=3, + SD, Student’s *t*-test). (B) WT EC sprouting is not affected by doxycycline simulation in 72 hr angiogenic sprouting assays (n=4 + SD, Student’s *t*-test). (C) qPCR shows no effect on mRNA expression of mesenchymal markers, endothelial markers, or LOXL2 in WT EC stimulated with doxycycline for 7 days (n=3).

**Figure S2. Effect of LOXL2 expression on angiogenic sprout quantity and length.** Quantification of an angiogenic sprouting assay reveals that knockdown of LOXL2 results in a reduction of sprouts per bead (A), but not in the length of the sprouts (B) (n=3, + SD, Student’s *t*-test) , whereas overexpression of LOXL2 results in an increase of the number of sprouts per bead (C), as well as the length of the sprouts (D) (n=4 + SD, Student’s *t*-test). *: *p*<0.05; **: *p*<0.01.

**Figure S3. LOXL2 does not affect expression of Snail in EC.** Knockdown (A) and overexpression (B) of LOXL2 does not affect Snail protein levels. (C) Snail expression is increased in EC after stimulation with TGF-β for 24 hrs.
